# Low Child Survival Index in a Multi-Dimensionally Poor Amerindian Population in Venezuela

**DOI:** 10.1371/journal.pone.0085638

**Published:** 2013-12-31

**Authors:** Julian A. Villalba, Yushi Liu, Mauyuri K. Alvarez, Luisana Calderon, Merari Canache, Gaudymar Cardenas, Berenice Del Nogal, Howard E. Takiff, Jacobus H. De Waard

**Affiliations:** 1 Dirección Regional de Salud Estado Delta Amacuro, Tucupita, Venezuela; 2 Laboratorio de Tuberculosis, Instituto de Biomedicina, Caracas, Venezuela; 3 Lovelace Respiratory Research Institute, Albuquerque, New Mexico, United States of America; 4 Escuela de Medicina José María Vargas, Universidad Central de Venezuela, Caracas, Venezuela; 5 Departamento de Pediatría, Hospital de Niños “J.M. de los Ríos”, Caracas, Venezuela; 6 Laboratorio de Genética Molecular, Instituto Venezolano de Investigaciones Científicas, Caracas, Venezuela; Tulane University School of Public Health and Tropical Medicine, United States of America

## Abstract

**Background:**

Warao Amerindians, who inhabit the Orinoco Delta, are the second largest indigenous group in Venezuela.  High Warao general mortality rates were mentioned in a limited study 21 years ago. However, there have been no comprehensive studies addressing child survival across the entire population.

**Objectives:**

To determine the Child Survival-Index (CSI) (ratio: still-living children/total-live births) in the Warao population, the principal causes of childhood death and the socio-demographic factors associated with childhood deaths.

**Methods:**

We conducted a cross-sectional epidemiological survey of 688 women from 97 communities in 7 different subregions of the Orinoco Delta. Data collected included socio-demographic characteristics and the reproductive history of each woman surveyed. The multidimensional poverty index (MPI) was used to classify the households as deprived across the three dimensions of the Human Development Index. Multivariable linear regression and Generalized Linear Model Procedures were used to identify socioeconomic and environmental characteristics statistically associated with the CSI.

**Findings:**

The average CSI was 73.8% ±26. The two most common causes of death were gastroenteritis/diarrhea (63%) and acute respiratory tract Infection/pneumonia (18%).  Deaths in children under five years accounted for 97.3% of childhood deaths, with 54% occurring in the neonatal period or first year of life.  Most of the women (95.5%) were classified as multidimensionally poor.  The general MPI in the sample was 0.56.   CSI was negatively correlated with MPI, maternal age, residence in a traditional dwelling and profession of the head of household other than nurse or teacher.

**Conclusions:**

The Warao have a low CSI which is correlated with MPI and maternal age.  Infectious diseases are responsible for 85% of childhood deaths.  The low socioeconomic development, lack of infrastructure and geographic and cultural isolation suggest that an integrated approach is urgently needed to improve the child survival and overall health of the Warao Amerindians.

## Introduction

According to last official Venezuelan Census (2001), the Warao people are the second largest Amerindian group in Venezuela [1]. They live mainly in the Orinoco River Delta; a fan of alluvial deposits within the coastal plain of eastern Venezuela subdivided by networks of fluvial and tidal channels [2], which is divided into three major geographical regions: upper (UOD), intermediate (IOD) and lower delta (LOD) [3]. The latest census showed that 81.4% of the Warao lived in the LOD, 15.2% in the IOD and 3.4% in the UOD [4].

The Warao live under precarious sanitary conditions [5-7] in approximately 364 villages [4] along the banks and on the islands of the Orinoco distributaries [3], generally with minimal infrastructure and very limited access to medical attention [5,7,8]. There are no roads or ground transportation in the Delta [5,7]. Recent studies have documented many of the severe health problems afflicting the Warao communities: respiratory tract infections(RTI) [9], tuberculosis [10,11], viral hepatitis [12,13] and HIV [14,15]. 

A critical problem is the lack of clean drinking water [6,7,16,17]. The Orinoco river, which Warao people have been drinking since ancestral times [8,18], has both organic [6] and inorganic pollution [19]. In addition, although most Warao still live in their original habitat [5,7], a sizable minority have recently experienced an accelerated process of acculturation [6], moving from their traditional villages within the Orinoco Delta to settle in “new” communities surrounding nearby urban centers such Puerto Ordaz and Tucupita, where they live in marginal communities that generally lack basic services [5,7,20]. 

Since most Warao people have no access to birth and death registration centers, there are very few statistics on mortality rates and causes of death in infants, children, adults and elderly. An investigation carried out from 1990-1993 [21] in a small geographical area estimated the total mortality rate per year in the Warao population as 12/1000 individuals, which was much higher than the mortality rate of 5/1000 for the general Venezuelan population during the same period [22]. That survey found that infectious diseases, principally gastroenteritis and tuberculosis, were the most common cause of death (46.5%). Other respiratory diseases, accounted for 8.2% of deaths, and 2.8% of deaths were associated with childbirth. The report also noted that in 60% of deaths, the individuals did not receive medical attention for the cause of death during the preceding six months [21]. As the mortality data from the prior study is now 21 years old, and there is no available data on infant mortality, we believed that another study was urgently needed. The objectives of the present study were to: 1) measure the child survival-index (CSI); 2) determine causes of child deaths; and 3) evaluate the socio-demographic factors associated with childhood deaths. 

## Methods

### Ethics Approval

This study was conducted in compliance with the Venezuelan Law on Indigenous People and Communities. Verbal and written permission to carry out the study was obtained from community leaders as well as the Office of Health Programs of Delta Amacuro State (DA) and SAOI -- the Office responsible for Indigenous Health in DA. Both offices approved the project and granted the authors permission to publish the results. Women participating in the study signed a written informed consent. Illiterate women signed by means of a thumbprint. The Venezuelan Ministry of Health and the Pan American Health Organization received written reports of the results of the study prior to submission for publication. 

### Population

**Figure 1:**
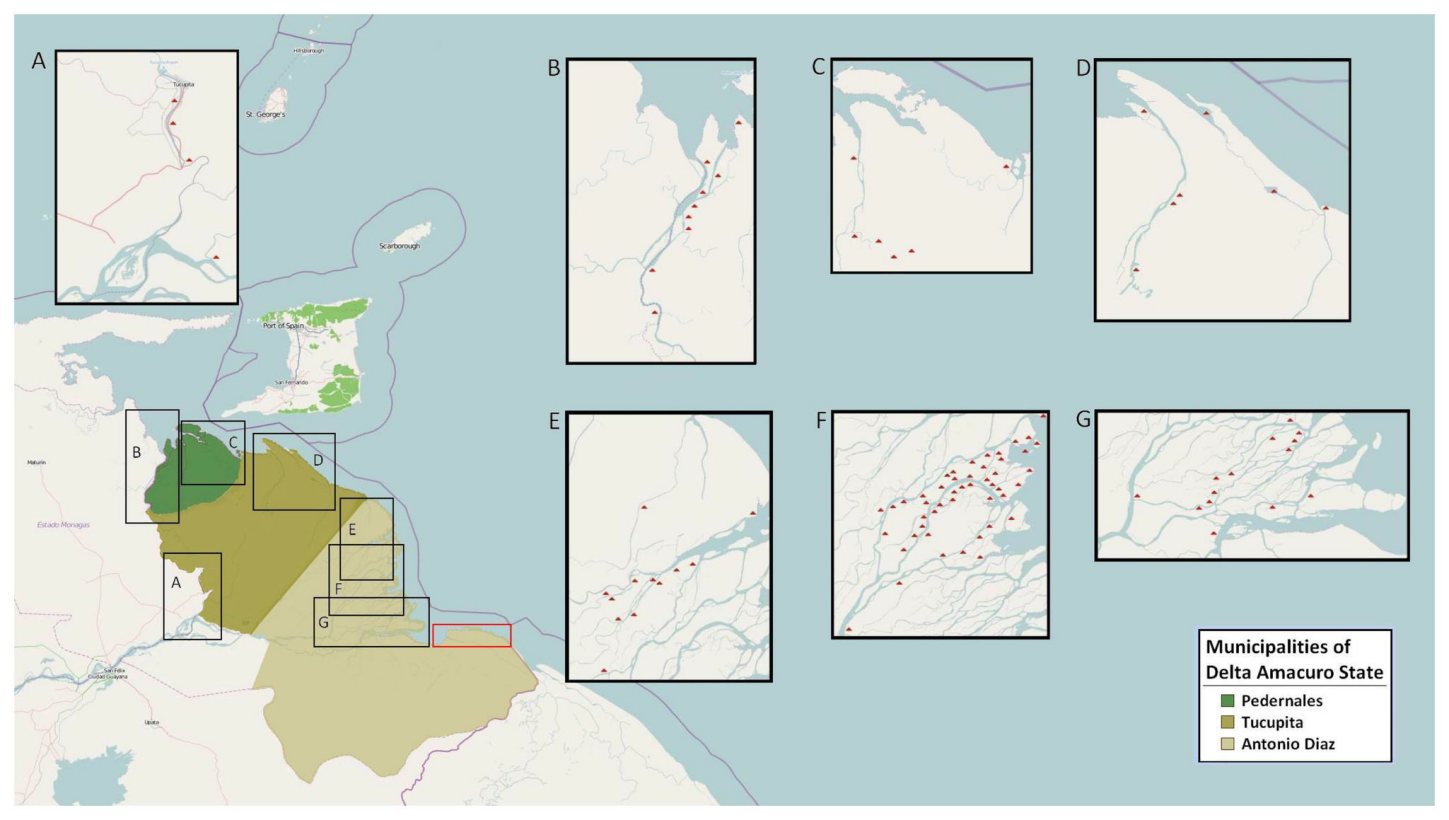
Map of the Orinoco Delta. Studied subregions are shown inside the black rectangles: Upper Orinoco Delta (*A*), Manamo Distributary (*B*), Capure distributary-Waranoko Surroundings (*C*), Mariusa Atlantic Coastline-Makareo Distributary (*D*), Nabasanuka surroundings (*E*), Guayo surroundings (*F*) and Curiapo surroundings (*G*). The red rectangle corresponds to Amacuro subregion which was not included in our survey. Triangles inside boxes correspond to the 97 studied communities.

In the 2001 Venezuelan National Census there were 26.080 Warao residing in DA, of which 48% were women[1,4]. The current epidemiological cross-sectional study was carried out in 97 communities (27% of the total Warao communities) [4] in seven different subregions of the LOD -- the original habitat of Warao tribe, -- and the UOD -- the principal area to which the Warao have recently migrated [7,20,23] (Figure 1). Due to logistical problems, Amacuro was the only subregion of the LOD excluded in our study. Inclusion of IOD was not within our objectives. The seven subregions that we studied contained an estimated 9305 women, 74% of the total female Warao population. 

### Epidemiologic Data Collection

**Figure 2:**
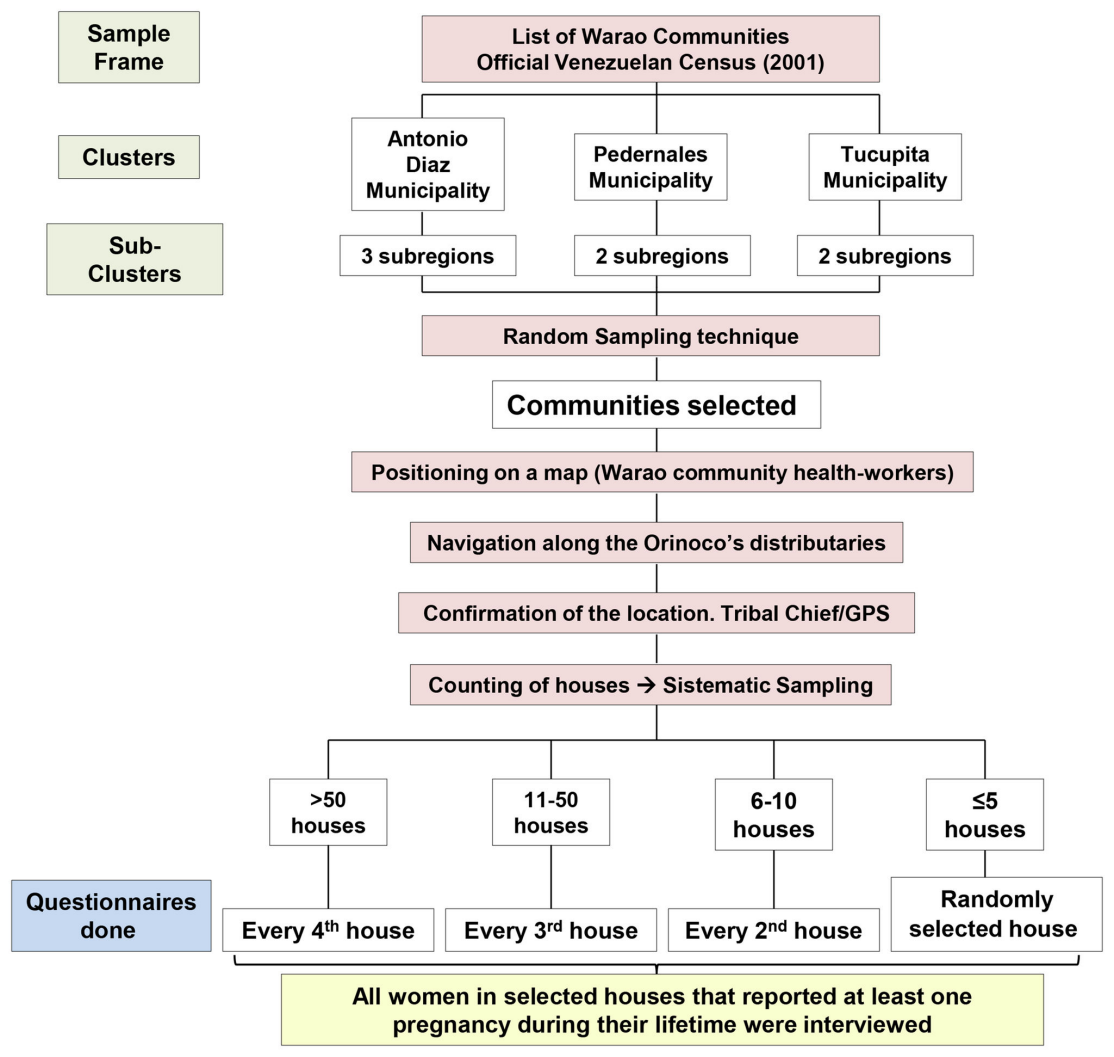
Flow Diagram. Default technique used to select interviewed-women of our sample in the communities of the Orinoco Delta.

The survey was conducted between October and November 2011, using a cluster sampling approach (Figure 2). The sampling was based on the three municipalities that have Warao population in the DA, using the list of communities appearing in the 2001 Venezuelan census [4] as the sample-frame. The proportion of communities selected from each municipality corresponded to the percentage of Warao communities that were located within the municipality (Table S1). The method for random selection of the communities to be sampled, and the geographic location and the size of the studied communities are given in the Methods S1 and Tables S2 and S3, respectively. To reach all the communities included in the study we traveled in small outboard boats along the Orinoco distributaries and streams for a total of 4825 Kilometers (2998 miles). 

Within each community a systematic sampling technique was used to select houses to be interviewed (Methods S1), and within each selected house, all women who reported at least one lifetime pregnancy were invited to participate. The only exclusion criterion was refusal to sign an informed consent.

All of the women in our study responded to a questionnaire with simultaneous translation to and from the Warao language by Spanish-Warao bilingual native interpreters. The questionnaire inquired about: 1) indicators of socioeconomic status, 2) level of literacy/education of the interviewed women and their husbands, 3) parity, and 4) the total number of children per woman according to the following categories: stillbirths, live-births, live-births who died and living children. Stillbirths were defined as any fetal death in which mothers could perceive human characteristics and shape in the fetus. In addition, we asked about any children who died in 2011, the year in which the study was carried out. The women were also questioned as to the causes of child deaths, which were classified according to the International Classification of Diseases (ICD-10) [24]. Maternal and paternal ages were determined from the national identity cards. The child survival-index (CSI) was defined as the ratio of still-living children to total-live births per Warao woman. 

The Multidimensional Poverty Index (MPI) was used to classify the households as deprived across the three dimensions of the Human Development Index (Health, Education and Living Standards). The original MPI [25,26] was modified slightly such that all the indicators were included except “nutritional status”, which was not assessed in the study. A detailed description of the dimensions and indicators used is further described in the Methods S2.

### Statistical Evaluation

The data was analyzed using Microsoft Excel, and the results reported as mean ±SD. The entire data collected in the study is provided as Datasets S1 and S2. Sex-specific differences between parents were analyzed using Pearson's chi-squared test. An error probability (P value) of <0.05 was considered significant. 

### Multivariable Linear Regression Analysis

We performed two multivariable linear regression analyses that evaluated associations between CSI and geographic regions, maternal, household and environmental risk factors using the t-test. The independent variables included both categorical and continuous variables (Table S4). 

The categorical variable “traditional household” was defined based on the characteristics of the family dwelling [5,7,18]: a one environment wooden house raised on stilts, without walls, with a ceiling made of palm tree leaves (*Manicaria saccifera* Gaertn.) and a wooden floor. Houses with additional architectural modifications and in which Spanish was spoken by the mother were regarded as non-traditional houses.

Two different Generalized Linear Model Procedures (PROC GLM) were used as equivalents of ANOVA. An overall F Test was used to examine the two models. The GLM models were used to decompose the variation in CSI (continuous dependent variable) by the sources of the different independent variables (Table S4). The effect of the variables regardless of order of introduction into the model was studied using Type III Sum of Squares (partial sum of squares). All statistical tests were performed in R version 12.4 [27] or SAS version 9.2 (SAS Institute, Cary, NC). 

## Results

### Characteristics of the Parents

In the course of the study 702 women were randomly selected, of whom only 14 (1.9%) were unwilling to be enrolled. A total of 688 women, belonging to 97 communities were interviewed, comprising approximately 5.5% of all Warao women living in the Venezuelan Orinoco Delta [1] and 7.4% of the female population of the 7 studied subregions[4]. The mean age of interviewed women was 34±12; 10.9% were under 19 years of age and 12.6% lived without a husband due to either the death or the abandonment of the spouse. The mean age of the fathers was 38±13. All the parents considered Warao to be their native tongue. Spanish proficiency was significantly lower (p<0.0001) in the women (51.6%) compared to their partners (66.6%) and illiteracy was significantly higher (58% versus 40%; p<0.0001). The household head was employed in only 16.9% of the homes. An employed individual was defined as any person over 16 years of age that spent at least 1 hour during the most recent week in "remunerative” job as a paid or self-employed worker. Only 10.5% of the women were living in traditional households. 

### Characteristics of Studied Communities

Of the 97 communities, 59% did not have an elementary school and 95% did not have a high school. Religious missions, which frequently provide education, were present in only 10% of the communities. Water treatment plants were present in only 8% of the communities, and 43.5% of the women collected and drank water directly from the river without prior treatment, while 37.4% drank collected rainwater when available. Only a small percentage drank water from communal taps (16.3%), domestic taps (1.9%) or bottled water (1.3%). None of the 97 communities had any kind of sewage system. The majority of the women (83.7%) had no area designated as a bathroom and a similar percentage (84.7%), practiced open-air defecation. Women commonly dropped litter on the ground (71.9%) or into the river (23.3%) and only 3.9% practiced open-air garbage burning. The majority of the communities had no fossil-fuel power plants (76%). There was no doctor in 93% of the communities and 85% lacked a primary healthcare facility. Although a precise measurement of economic indicators was not within the initial objectives of this study, it is apparent from the data collected (Tables 1 and S5) that the Warao live in conditions of poverty. 

**Table 1:** Warao Women’s Access to basic services.

**Without access to**	**Frequency (%)**	***95% CI***
Elementary School	209 (30.4)	27.1-33.9
High School	562 (81.7)	78.6-84.4
Religious Mission	531 (77.2)	73.9-80.2
Fossil Fuel Power Plant	341 (50.4)	45.4-54.1
Water Treatment Plant	594 (86.3)	83.6-88.7
Sewage System	688 (100)	-
Primary Healthcare Institute	443 (64.4)	60.7-67.9
Medical Doctor	545 (79.2)	76-82.1

### Multidimensional Poverty Index (MPI)

**Figure 3:**
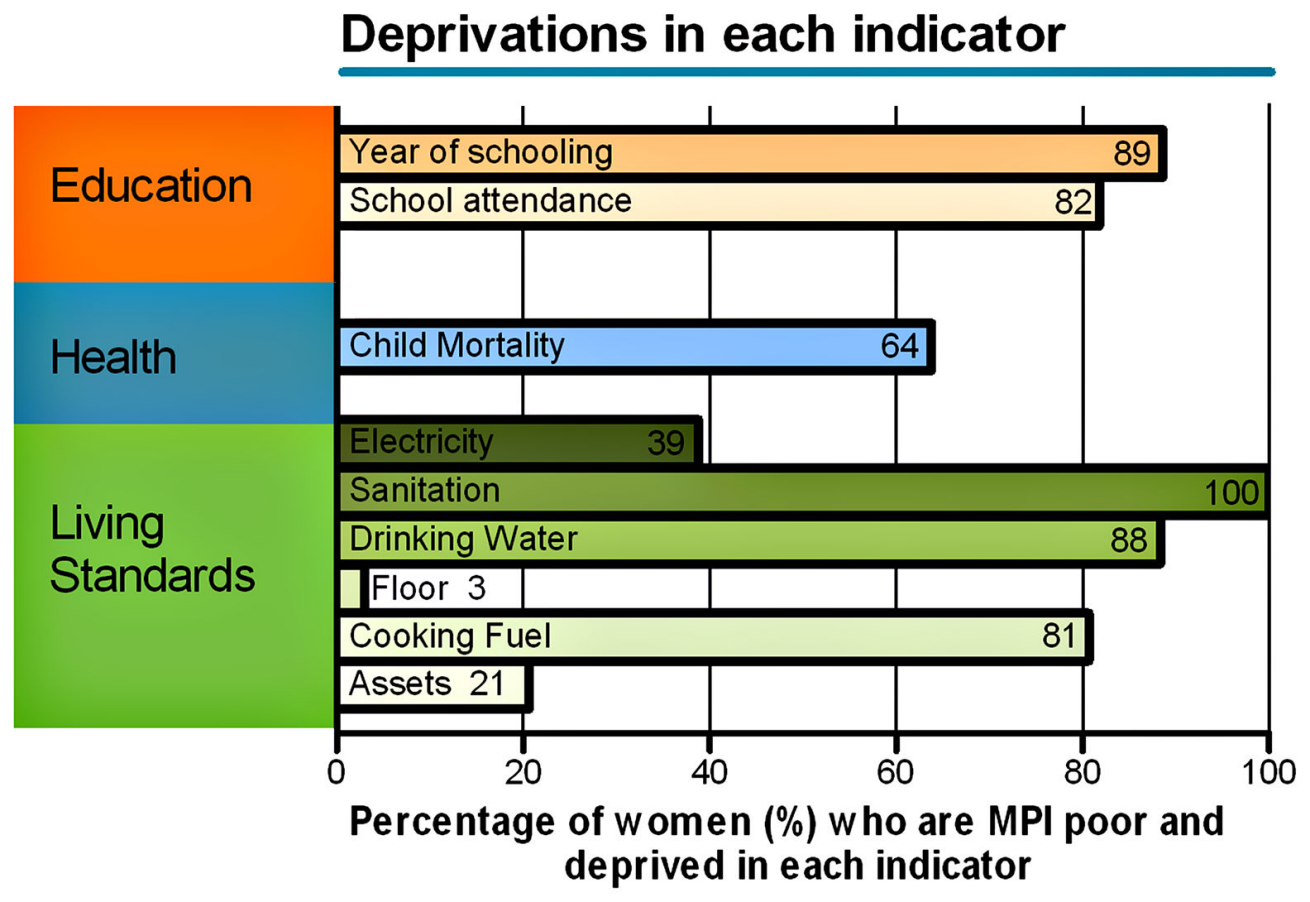
Percentage of women who are deprived in each indicator. Percentage of women deprived in each indicator of the three dimensions of the Human Development Index (Education, Health and Living Standards). The indicator “Nutritional status” was not assessed in the study.

Most of the women, 95.5% (657/688), could be described as multidimensionally poor, as they were classified as deprived in at least 33.3% of weighted indicators (Figure 3). Nutritional status, however, often included in the MPI, was not assessed in our study. The mean intensity of MPI among households classified as MPI-deprived was 0.59 ± 0.11, while the mean for the entire sample was 0.57±0.14. The highest mean MPI per household was found in the Tucupita municipality (0.62 ±0.1) followed by Pedernales (0.60 ±0.1), while Antonio Diaz had the lowest mean MPI score (0.56 ±0.14) (Figure S1). The general MPI in our sample was 0.56 (Table 2).

**Table 2:** Multidimensional Poverty Index among municipalities.

Dimensions	Indicators	Frequency of Deprivation (%)				
		Municipalities			Total		
		Antonio Diaz	Pedernales	Tucupita	
Education					
	Years of Schooling	429 (86)	95 (97)	86 (95)	610 (89)
	Child School attendance	400 (80)	79 (81)	84 (92)	563 (82)
Health					
	Child Mortality	307 (62)	71 (72)	61 (67)	439 (64)
	Nutrition*	-	-	-	-
Standard of Living					
	Electricity	201 (40)	24 (25)	42 (46)	267 (39)
	Sanitation	499 (100)	98 (100)	91 (100)	688 (100)
	Drinking Water	441(88)	97 (99)	70 (77)	608 (88)
	Floor	0 (0)	5 (5)	16 (18)	21 (3)
	Cooking Fuel	403 (81)	77 (79)	75 (82)	555 (81)
	Assets (n=644)	73 (15)**	25 (26)	35 (38)	133 (21)
Total MPI Deprived		470 (94)	97 (99)	90(99)	657 (95.5)
Average		0.559	0.601	0.623	0.573
Average intensity of MPI poor people		0.58	0.604	0.627	0.59
General MPI		0.546	0.598	0.621	0.563

^*^ “Nutrition” was a missing indicator in our sample

^**^ ”Assets” was a missing indicator in 44 women of Antonio Diaz Municipality

### Child Survival

In the study sample, 3,937 live births were recorded, of which 2,692 (68.4%) were still living and 1,245 (31.6%) had died from various causes. The average CSI was calculated as 73.8%±26. The women interviewed reported a total of 196 stillbirths (0.28/woman), translating into 1 per 20.1 live births. The range of dead children per mother was 0-15, and the range of still-living children per mother was 0-12. The study identified 28 Warao children less than five years of age who had died between January-November of 2011. The CSI values among different geographic locations (municipalities and subregions) and mother’s age groups are shown in Tables 3, S6 and 4, respectively. 

**Table 3:** Distribution of sample and child survival-Index among Municipalities of Delta Amacuro State.

**Municipality**	**Frequency (%)**	Total Female Population*	**% Represented by sample (*95% CI*)**	**Dead children (%)**	**CSI (Mean ± SD)**
Antonio Diaz	499 (72.5)	8875	5.6 (5.2-6.1)	29.3	75.3 ± 26
Tucupita	91 (13.2)	2301	3.9 (3.2-4.8)	39.0	69.4 ± 29
Pedernales	98 (14.2)	1346	7.3 (6-8.8)	35.1	70.6 ± 23.6
TOTAL	688 (100)	12522	5.5 (5.1-5.9)	31.6	73.8 ± 26

^*^ Venezuela’s National Census (2011) was used as a reference [4].

**Table 4:** Child survival-Index in relation with Warao women age groups.

**Age of Interviewee**	**Frequency (%)**	**Child Survival-Index (Mean ± SD)**	**Live Births (Mean ± SD)**
≤ 20years	75 (10.9)	90 ± 24.4	1.7 ± 0.8
21-30 years	249 (36.2)	75.8 ± 25.4	4 ± 2.2
31-40 years	191 (27.8)	74.6 ± 23.6	6.7 ± 2.8
41-50 years	99 (14.4)	63.8 ± 26	8.7 ± 3.4
51-60 years	45 (6.5)	60.7 ± 24.5	8.8 ± 4
>60 years	29 (4.2)	64.4 ± 25.3	9 ± 3.3
TOTAL	688 (100)	73.84 ± 26	5.7 ± 3.5

**Figure 4:**
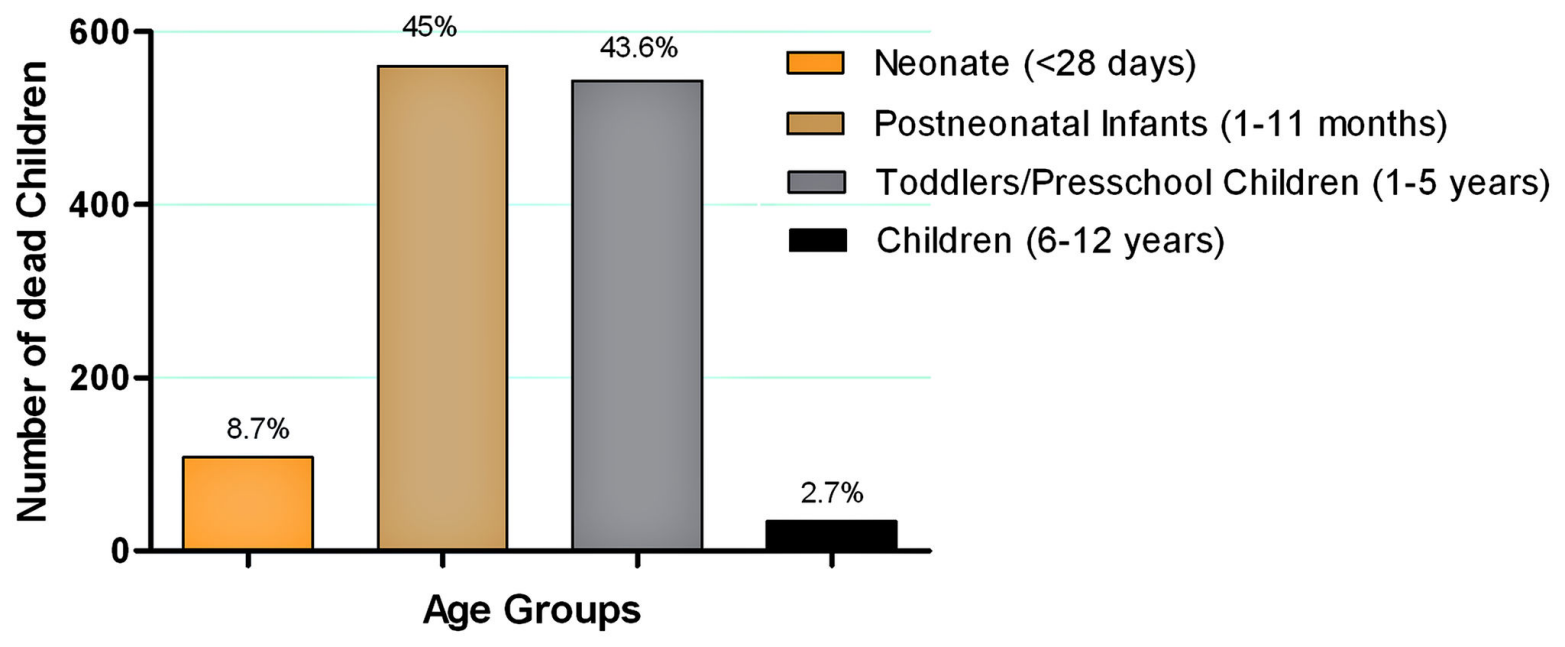
Deaths among Warao live-births by age groups.

**Figure 5:**
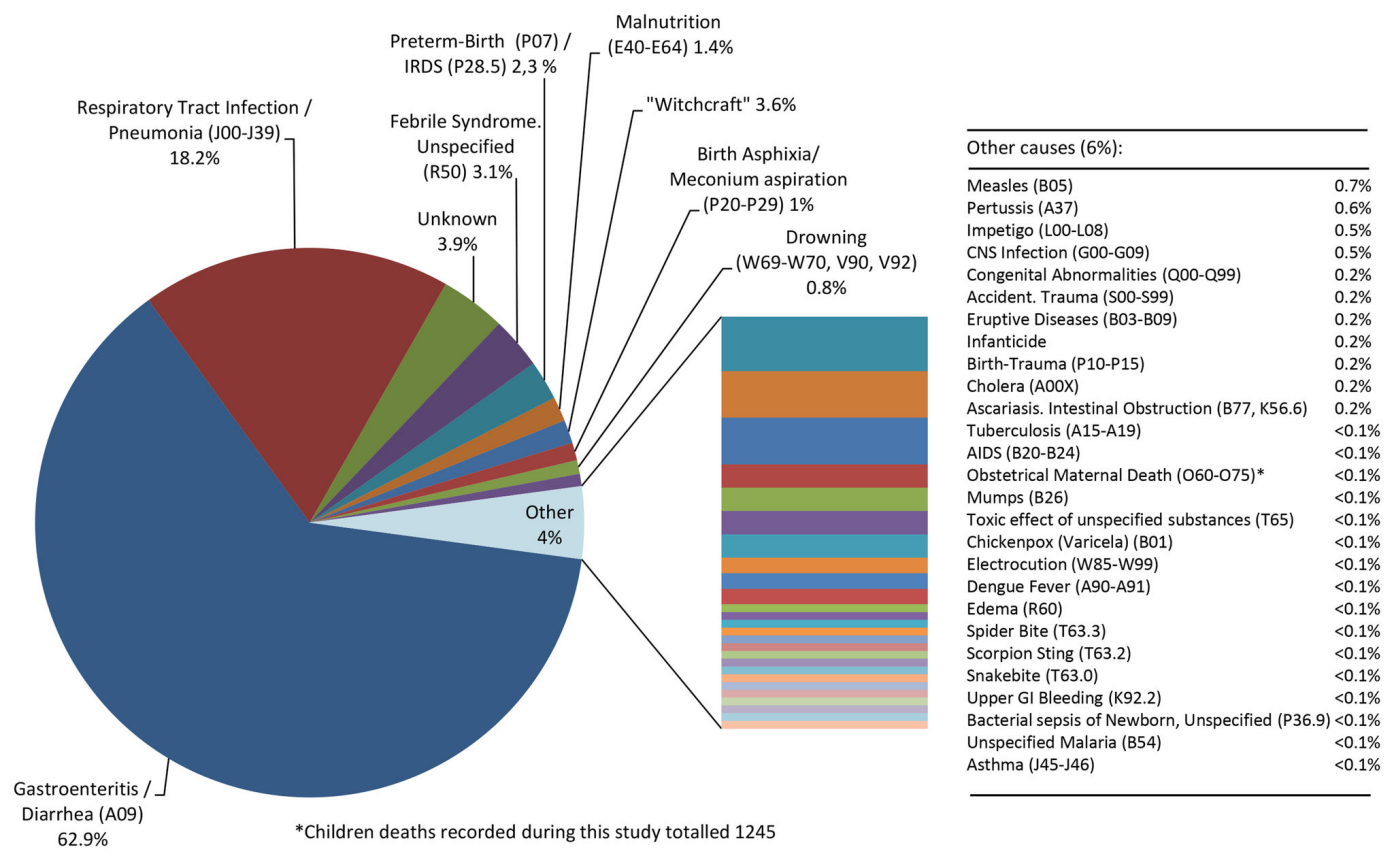
Deaths among Warao live-births by cause. Causes of death were classified according to the International Classification of Diseases (ICD-10)^24^, although in some conditions the reported cause of death was unlikely to be the cause. Codes for diseases are shown in parentheses.

Of the 1245 childhood deaths, 97.3% were under five years of age (Figure 4) and more than one-half (56.3%) of all deaths were males. Infectious diseases were responsible for 85% of the deaths, with diarrhea (63%) and acute respiratory tract infections/pneumonia (18%) as the principal causes of death reported among live births. A variety of other causes accounted for the remaining 19% of deaths (Figure 5). Determination of the cause of death was based on the information provided by interviewed mothers, as death certificates were rarely available. The causes of death among different age groups are presented in Figure S2. 

### Determinants of Child Survival

In a first model of multiple linear regression, CSI was significantly and negatively correlated with maternal age, profession of the household head being other than nurse or teacher, maternal Illiteracy, having been born in Tucupita Municipality and living in a community without a water treatment plant or elementary school (Table 5). In a second model of multiple linear regression analysis in which subregions were included as a variable instead of municipalities, only the first three variables mentioned above were significantly associated with CSI (Table S7). In both model the regression coefficients were small, except for the variable “profession of the head of the household” (-0.14; SE 0.04) and the cumulative coefficient of maternal age (-0.006 SE 0.0008) over 20-30years.

**Table 5:** Multivariable linear regression analysis. Model 1: Association of CSI with socioeconomic factors.

**Variables**	**Subcategory**	**Children Survival**		
		**Coeff (SE) †**	***T* value**	***P* value**
**Mother’s Place of Residence**				
	Antonio Diaz Municipality	Reference		
	Pedernales Municipality	-0.02 (0.03)	-0.46	0.65
	Tucupita Municipality	-0.06 (0.03)	-1.95	0.05 *
**Mother’s Characteristics**				
	Age	-0.006 (0.0008)	-7.12	<0.0001 *
	Illiteracy	-0.06 (0.02)	-2.74	0.006 *
**Living in a community**				
**without access to**				
	Elementary school	-0.07 (0.03)	-2.1	0.04 *
	Primary healthcare institution	-0.04 (0.04)	-1.15	0.25
	Water treatment plant	-0.09 (0.04)	-2.5	0.01 *
	Medical doctor	-0.03 (0.04)	-0.68	0.49
	Religious mission	0.02 (0.04)	0.6	0.58
**Size of the Community**		0.02 (0.01)	1.8	0.07
**Household Characteristics**				
	Profession of the household head	-0.14 (0.04)	-3.4	0.0008 *
	(being other than nurse or teacher)			
	Number of People per house	0.002 (0.002)	0.9	0.35
	Absence of water treatment practices	0.009 (0.03)	0.33	0.74
**Others**				
	Presence of an outboard motor	0.02 (0.02)	0.77	0.44
	Main water source (river water)	0.006 (0.03)	0.24	0.81

^†^ Negative sign (coefficient) denotes inverse association of the independent variable with CSI

^*^ Statistically significant *p*-value <0.05

Residual standard error: 0.2407 on 628 degrees of freedom (46 observations deleted due to missingness)

Multiple R-squared: 0.1721 / Adjusted R-squared: 0.1524

F-statistic: 8.705 on 15 and 628 DF / p-value: < 2.2e-16

When additional variables were included in two different Generalized Linear Models that considered all variables simultaneously, CSI remained significantly correlated only with maternal age, MPI, profession of the head of household other than nurse or teacher and residence in a traditional household (Tables 6 and S8). Model Fitness Information of both models is specified in Table S9. 

**Table 6:** Generalized Linear Model predicting determinants of Child Survival Index (CSI). Model 2.

**Variables**		**Degrees of Freedom**	**F Statistic**	***p*-value**
**Municipality of residence**		2	0.16	0.86
**Mother’s Characteristics**				0.32
	Age	1	15.78	<0.0001*
	Illiteracy	1	0.00	0.99
	Parity	1	2.92	0.08
**Living in a community**				
**without access to**				
	Elementary School	1	2.97	0.09
	Primary Healthcare facility	1	1.34	0.25
	Water Treatment Plant	1	0.2	0.66
	Medical Doctor	1	1.00	0.32
	Religious mission	1	2.03	0.15
**Size of the community (number of houses)**		1	2.09	0.15
**Household Characteristics**				
	Profession of the household head	1	3.94	0.04*
	(being other than nurse or teacher)			
	Number of People per house	1	1.65	0.19
	Father’s Presence	1	0.36	0.55
	Traditional House	1	8.59	0.003*
	MPI	1	152.97	<0.0001*

^*^ Statistically significant. *p*-value <0.05

## Discussion

The Orinoco is South American’s second largest river. At its delta it branches into a multitude of rivers and waterways that separate hundreds of islands in a vast territory of 40,200 square kilometers located in Venezuela’s Delta Amacuro State. It has a very low population density - 3.8people/km^2^ overall, but excluding the 100,000 non-Amerindians living in or near the capital of Tucupita, the density is only 0.5/ km^2^ [1]. The Warao likely moved to this remote and ecologically difficult region hundreds of years ago to escape conquest by other tribes [5], and the strategy seemed to have worked, as they are the second largest of some twenty tribes in Venezuela, with over 20,000 members [4,5]. The same geographic isolation has also limited the intrusion of non-Amerindian society. A constant presence of non-Amerindian culture came only in the 1950’s, with the establishment of Church missions in a few communities [5,8]. Still 60 years later, aside from a very few relatively large communities (500 - 1000 people) that have grown around missions, most of the communities do not enjoy even minimal benefits of development -- only seven of the 97 communities sampled had access to a medical doctor and just 15 had a primary health facility. The communities in the lower delta are accessible only by traversing the many channels and distributaries in small boats. There are few signs posted, and the maps are inadequate, so the pilots navigate by recognizing the islands and tree patterns as landmarks. Using a powerful speedboat, the trip from the capital to some of the distant larger communities around church missions, such as Nabasanuka, takes 4 - 5 hours, but to reach the most distant, most traditional and isolated communities can take up to 8 - 10 hours. In the dugout canoes used by the Warao, the trip from these communities to the capital, Tucupita, where there are secondary medical facilities and a hospital, can take several days (Villalba JA personal communication). The 15 primary medical facilities in the Delta are not equipped to handle a very sick infant, and most do not have on-call doctors [7]. From the observations of one of the authors (JdW) who has worked for many years in the Delta, it was clear that infant mortality among the Warao was extremely high and development and socioeconomic status extremely low, but there were no recent estimates or studies documenting child survival in the region. 

### Multidimensional Poverty

The MPI (Multidimensional Poverty Index) was developed in 2010 by the Oxford Poverty & Human Development Initiative and the United Nations Development Program to quantify deprivations in very rudimentary services and core human functioning beyond income-based classifications. An overwhelming majority of our sample (95.5%) were classified as deprived in more than a third of the weighted indicators. When compared with data for 104 developing countries, only Niger has a higher MPI (0.642) than the Warao in our sample (0.564) [28]. There is no available data about MPI in Venezuela as a whole, but a recent report of the United Nations Development Program classified Venezuela in the high human development category (HDI=0.748), ranking 71 out of 187 countries evaluated [29]; suggesting that the Warao have a much lower socioeconomic status than most non-indigenous Venezuelans. 

### Causes of Child Deaths

Using a random proportional approach to sample the entire Warao population, we traveled nearly 3000 miles on the waterways of the delta to interview 688 women in 97 villages. We obtained reports of 3937 live births, an average of 5.7 per woman interviewed, with 196 stillbirths or 0.28/woman. The average CSI was only 73.8%±26. Anthropologists have reported that Warao women expect that up to half of their children will die during their lifetime[20].

A previous survey in 1990-1993 covering only a limited region of the delta, determined that 55.3% of all Warao deaths occurred in children less than five years of age[21]; our survey found that deaths under 5 years represented 97.3% of all the deaths in children less than twelve years. Neonatal (<28 days) and post neonatal deaths (1 month-1year of age) accounted for 53.7% of the total, while deaths in toddlers and preschool children (1-5years) represented 43.7%, and just 2.6% of deaths occurred in children older than 5 years. As previously reported [6], the majority of the deaths (63%) were caused by gastroenteritis or diarrhea. The overwhelming majority of the interviewed-women (86.3%) lived in communities without water treatment plants, collected untreated drinking water directly from the river (43.5%), did not have a designated place used for defecation (83.7%), and none of the studied communities had any kind of sewage system. While this does not establish a direct relationship between water source and gastrointestinal illness, and breast feeding is universal in the Warao (Franco C, Del Nogal B, unpublished data), both access to drinking water and sanitation coverage have been linked with lower mortality in children under 1 and 5 years of age in a study evaluating data from 21 Latin American countries [30].

The second most frequently reported cause of death (17%) among Warao Children was acute respiratory tract Infection or pneumonia. A recent study documented a high prevalence of pneumococcal carriers among the Warao children [31] as has been found in other indigenous populations [32], which would imply a high risk for invasive pneumococcal disease, such as seen in other native populations in New Zealand [33] and the United States [34]. It is possible that many infant deaths could be prevented if high coverage could be achieved in very young Warao children with rotavirus, pneumococcal and *Haemophilus influenzae* type B vaccines. While there were also deaths attributed to measles, mumps and pertussis, these were very few, with no evidence of large outbreaks. Although these diseases are covered by the Venezuelan Immunization program [35], a prior study found a low vaccine coverage in Warao children [9]. However there are few possible explanations why these very contagious infections were uncommon: the population is largely divided into small isolated communities so outbreaks do not spread; the vaccination coverage may have been underestimated; the vaccinations may still have been relatively effective for herd immunity; or the cause of the illnesses, as interpreted from the descriptions given by the mothers interviewed, was inaccurate. Because the study didn’t assess nutritional status, it is not possible to determine whether poor nutrition contributed to the susceptibility to infections, but malnutrition accounted for just 1.4% of deaths, suggesting that severe malnutrition is not highly prevalent in the population. 

Official reports of indigenous populations in several countries, including the United States [36,37], Canada [38] and Australia [39], have found infant mortality rates considerably higher than in the non-indigenous populations. The principal causes of infant deaths among Native American in the US were congenital malformations, sudden infant death syndrome and disorders related to short gestation [40]. Although 92% of childhood deaths in the Warao sample were reported to have occurred after the neonatal period, there were 108 deaths during the first month of life. Of these, 28% were caused by infant respiratory distress syndrome (IRDS) associated with preterm births (Figure S2), 12% were caused by birth asphyxia or meconium aspiration and 20% by unknown causes. There were also many stillbirths (1 per 20.1 live-births). Studies in different geographical settings have associated stillbirths and pregnancy losses with deficient or absent prenatal care [41,42], suggesting that providing the Warao with prenatal care and assisted deliveries, although logistically challenging, could reduce the number of stillbirths and neonatal deaths.

Although we found evidence of gender inequality, with both literacy and Spanish proficiency significantly lower in mothers than fathers, we also found three cases of Infanticide of boys in families who desired girls. Anthropologists have reported that girls are the economic and spiritual center of the Warao family life, and selective male infanticide has been previously described, especially in children with malformations [20]. We also found slightly more male (56.3%) than female childhood deaths, which could perhaps be related to preferential maternal care for female children, especially newborns; however, this is merely speculation, and additional studies are needed to investigate this topic. 

### Determinants of Child Survival

In our statistical models we found many variables that were individually associated with a lower CSI, but when considered together in an additive model, the only highly significant associations were with: 1) MPI; 2) maternal age; and 3) traditional home; with borderline associations for profession of the head of the household other than nurse or teacher, parity and lack of a primary school in the village (Table 6). When adjusting for maternal age and MPI in the model, the negative individual associations between CSI and mother’s illiteracy, absence of elementary school and water treatment plant in the communities were no longer significant. Perhaps several of these latter variables lost significance because they are highly correlated with low MPI or residence in the more traditional villages, which are the most remote and underdeveloped. Given the high percentage of childhood deaths associated with gastrointestinal disease, it is surprising that there was not an independent association with villages having a water treatment plant; some of these plants, however, were only recently installed so beneficial effects may not yet be manifested; and they supply domestic water to only two communities. In addition bathing and swimming in the river are universal habits in the Warao [6]. We hypothesize that the borderline association of higher infant survival with the head of household being either a teacher or a nurse (p=0.04) could be because these families have more education, are more likely to have familiarity and contact with services in the capital Tucupita, and are also more likely to have access to the transportation necessary to get there for pre and postnatal health care; Interestingly this variable had the highest regression coefficient in out two models, which indicates that this variable had the greatest influence on the CSI in our models of regression analysis. 

An association of infant mortality with high MPI might be expected; however, the highly significant negative association with maternal age (Tables 5, 6, S7 and S8) is intriguing. The mean CSI was highest in women under 20 years (90±24.4), and lowest in women 50 - 60 (60.7+24.5), while still-fertile women (21-40 years) had a mean CSI between the two extremes (75.3±24.6). The younger women would be expected to have had fewer live-births (1.7±0.8) and fewer years of childcare, and some of their children would not have reached the age of 5 years after, which the infant mortality rate declines dramatically, but the association with maternal age was much stronger than the borderline negative association of CSI with parity (p = 0.08). The regression coefficient for the variable "age" in our model suggests that a 50 years old woman would have approximately a decrease of 18% in the CSI compared to a 20years old woman. There is some controversy in the literature about the effect of maternal age on child survival. A study in India of two culturally distinct populations showed that child mortality was lower in younger women in both groups, but Infant mortality was higher in older women in only one of the two cohorts [43]. In Ivory Coast, young women with no education appear to have lower child mortality than older women, in both urban and rural areas, but in Ghana the child mortality rates for younger and older women were similar [44]. We speculate that the strong association in the Warao could be because younger mothers are more healthy at the time of their first birth; have more time for childcare; or live in a less constrained and/or less traditional environment than older women; or are more accustomed to using health care services. Another possible explanation is that the availability of health care services has improved over the 10-20 years. The association is interesting but unexplained, and deserves to be investigated further. 

Although the association of CSI with municipality was not significant in the additive model, there were regional differences in the CSI (Tables 3 and S6); however, this may be due to common cause variations. 

### Underreporting and Child Survival

The low CSI found in this survey does not correspond to the official data available, which is not unexpected, as the Warao’s geographic and cultural isolation precludes accurate statistical tracking of births and deaths. Two examples illustrate the official underestimates of childhood deaths in the Warao. In our survey we collected reports of 28 deaths in children under five years of ages in the 11 months between January and November of 2011. As the survey included only 688 women or about 7% of the female Warao population in the regions that were sampled, a conservative estimate of the total deaths of Warao children under five in these regions might be five times greater, or 140. However, the Office of local Epidemiology of the DA reported just 107 deaths in the same age group during the period [Villalba, J, personal communication]; this tally included all childhood deaths in the entire population of 124,067, of which 79% are non-Amerindian [1]. In another example, the women interviewed in the Pedernales Municipality reported 6 deaths under five years of age during the study period, however, the local epidemiology office recorded only 2 childhood deaths. Official underreporting of childhood deaths has been found in other Amerindian populations, where childhood deaths are considered normal sad events in the life of the family; a study in the state of California estimated that deaths of American Indian children from 1979 through 1993 were three to four times greater than recorded on death certificates [45].

In summary, we have documented that the Warao living in the delta have, on average, a very low CSI, although there are important subregional differences. The low CSI is statistically associated with a multiply deprived socioeconomic status, older maternal age, and living in traditional dwellings. While ameliorating the socioeconomic depravation in the Warao community of over 20,000 individuals would be a long term goal, the CSI could likely be improved over the medium term with targeted interventions such as broader and more comprehensive vaccine coverage and also improved access to prenatal and perinatal care. Although the geography and population distribution in the delta make any population-wide interventions logistically challenging, these are basic services that should be provided. Several studies have documented very high rates of tuberculosis among the Warao [10,11] and very recent reports have described a growing problem with HIV [14,15]. Although only few childhood deaths from these two scourges were reported in our study, it is likely that in the near future they will together be a more common cause of childhood mortality.

## Limitations

Our study has several limitations, especially in our analysis of statistical associations with CSI. We did not adjust for some potentially important confounders such as birth weight and prenatal health history; it was difficult or impossible to obtain accurate information on these variables from the interviewed subjects because very few births are attended by experimented physicians or nurses. We also failed to ask about other possible factors such as deaths in multiple pregnancies and deaths by birth order. We could not accurately assess nutritional status, because of the lack of criteria for Amerindian populations. Although we had reports of 28 deaths in children under 5 years of age during the 11 months immediately prior to our study, we only recorded the age of children when they died, then we do not have the total number of children born during this period (children under 11 months of age); thus, we have no way of converting these 28 deaths into an estimate of the child mortality rate. Because of the many villages to be sampled and the general homogeneity of the Warao population, we chose the systematic sampling method to try to efficiently obtain a representative cohort; however, we are aware that this widely used sampling technique could possibly introduce an unintentional bias due to the lack of information about the socioeconomic structure of the sampled villages. Point estimates of CSI were unweighted to account for differences in the ultimate probability of selection in the sampling process, and therefore should be interpreted with caution since they are not 100% precise. Moreover, standard errors and 95% confidence intervals for point estimates and regression coefficients were estimated assuming that simple random sampling was used to select survey respondents instead of a multi-stage sampling process that was actually used which would require empirically-estimated standard error estimation methods to account for correlated data structure within sample clusters.  Because of this, 95% confidence intervals about point estimates are likely narrower than they should be, and the significance of regression coefficients may be overstated. Therefore, the results should accordingly be treated with caution. This study was a first attempt to estimate both CSI and MPI in this diffuse and remote population. While we believe our data represents accurate approximations on a population level, the absolute rates for the different variables are likely not completely precise, and we may have missed regional or local differences. A multilevel analysis or a Cox proportional hazard model should be applied in future studies to evaluate both individual and community-level factors affecting the CSI, perhaps through more intensive local studies rather than the more comprehensive global approach pursued in this study.

## Conclusion

Although Venezuela overall could be close to meeting some of the UN Millennium development goals [46], this study and others [9-11,14,15,46], show that the Warao population represents a pocket of poverty and underdevelopment in which targets for six of the eight Millennium development goals (Goals 1, 2, 3, 4, 6 and 7) [47] are far from being achieved. Ethnic-specific approaches adapted to the difficult geography will be needed to decrease preventable causes of childhood mortality in this highly vulnerable population.

## Supporting Information

Dataset S1 **Data of child deaths, CSI determinants, and characteristics of the family dwelling.**
(XLS)

Dataset S2 **Data of the MPI parameters.**
(XLS)

Figure S1 **MPI among different municipalities in studied sample.** Regional differences between pairs were analysed using Mann-Whitney U test; p-values are shown in brackets.
(TIF)

Figure S2 **Causes of Death in Warao Children regarding different age groups.** Neonates, <28 Days (*A*), Post-neonatal infants, 1-11 months (*B*), Toddlers and Preschool Children, 1-5 years (*C*), and children between 5 and 12 years (*D*). Causes of death were classified according to the International Classification of Diseases (ICD-10)^24^, although in some conditions the reported cause of death was unlikely to be the cause. Codes for diseases are shown inside parentheses.
(TIF)

Methods S1 **The detailed methods of sample selection (communities and houses selection) are described in Methods S1.**
(DOC)

Methods S2 **The detailed MPI definition used in this study is described in Methods S2.**
(DOC)

Table S1 **Number and proportions of communities selected among different municipalities.**
(DOC)

Table S2 **List of communities selected according to geographic location (Municipality).**
(DOC)

Table S3 **Distribution of the sample regarding size of the evaluated communities.**
(DOC)

Table S4 **Variables included in the different models.**
(DOC)

Table S5 **Characteristics of communities among different geographical regions (Basic Services).**
(DOC)

Table S6 **Distribution of sample and child survival-Index among different subregions of the Orinoco Delta.**
(DOC)

Table S7 **Multivariable linear regression analysis. Model 2: Association of CSI with socioeconomic factors.**
(DOC)

Table S8 **Generalized Linear Model predicting determinants of Child Survival Index (CSI). Model 1.**
(DOC)

Table S9 **Model Fitness Information of generalized linear model procedures.**
(DOC)
