# Supplementary figures and images for: Low Child Survival Index in a Multi-Dimensionally Poor Amerindian Population in Venezuela
Source: PLoS One. 2013 Dec 31;8(12):e85638. doi: 10.1371/journal.pone.0085638 (PMC3877389; doi:10.1371/journal.pone.0085638)

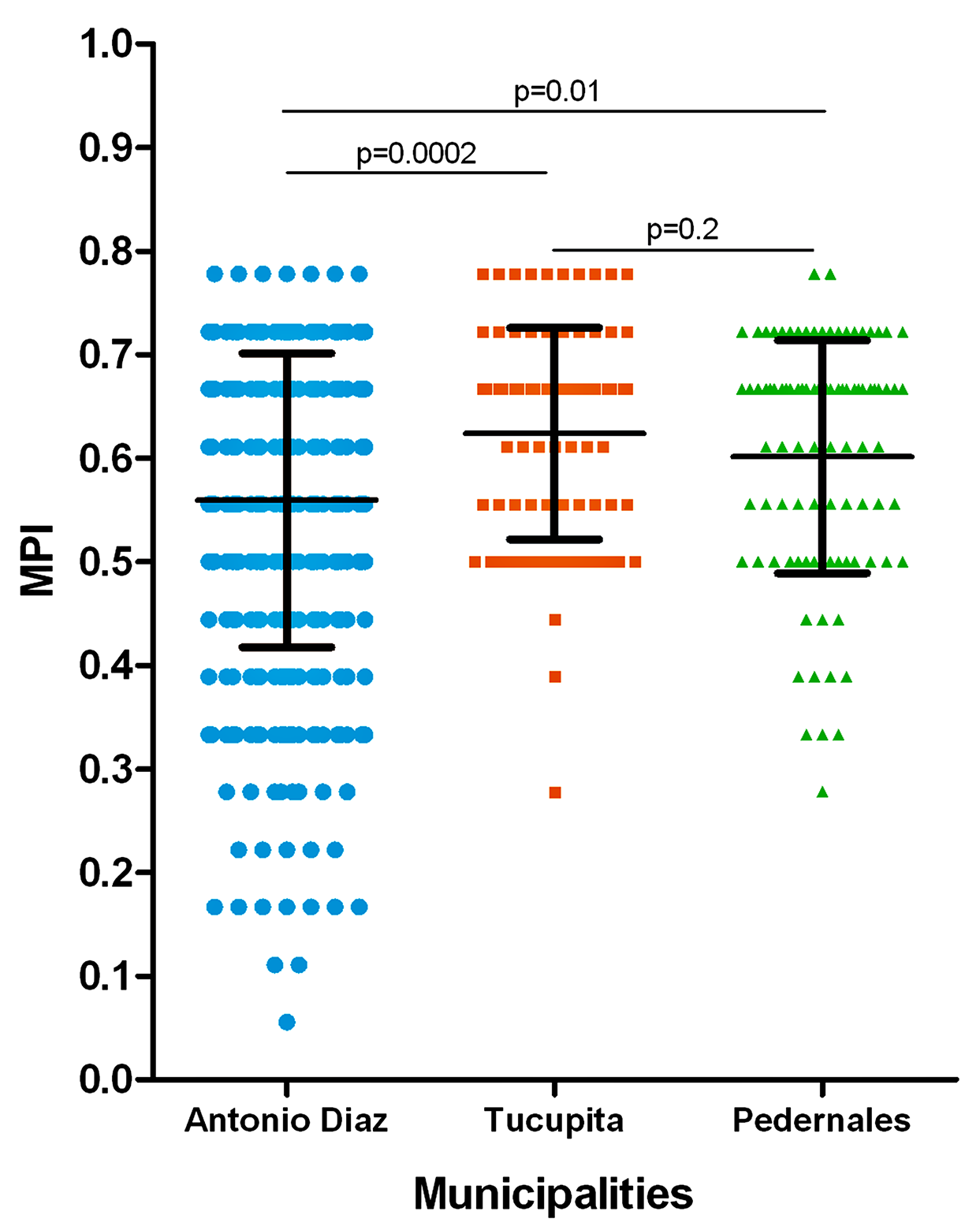

Supplement: Figure S1 — MPI among different municipalities in studied sample. Regional differences between pairs were analysed using Mann-Whitney U test; p-values are shown in brackets. (TIF) [file pone.0085638.s003.tif]

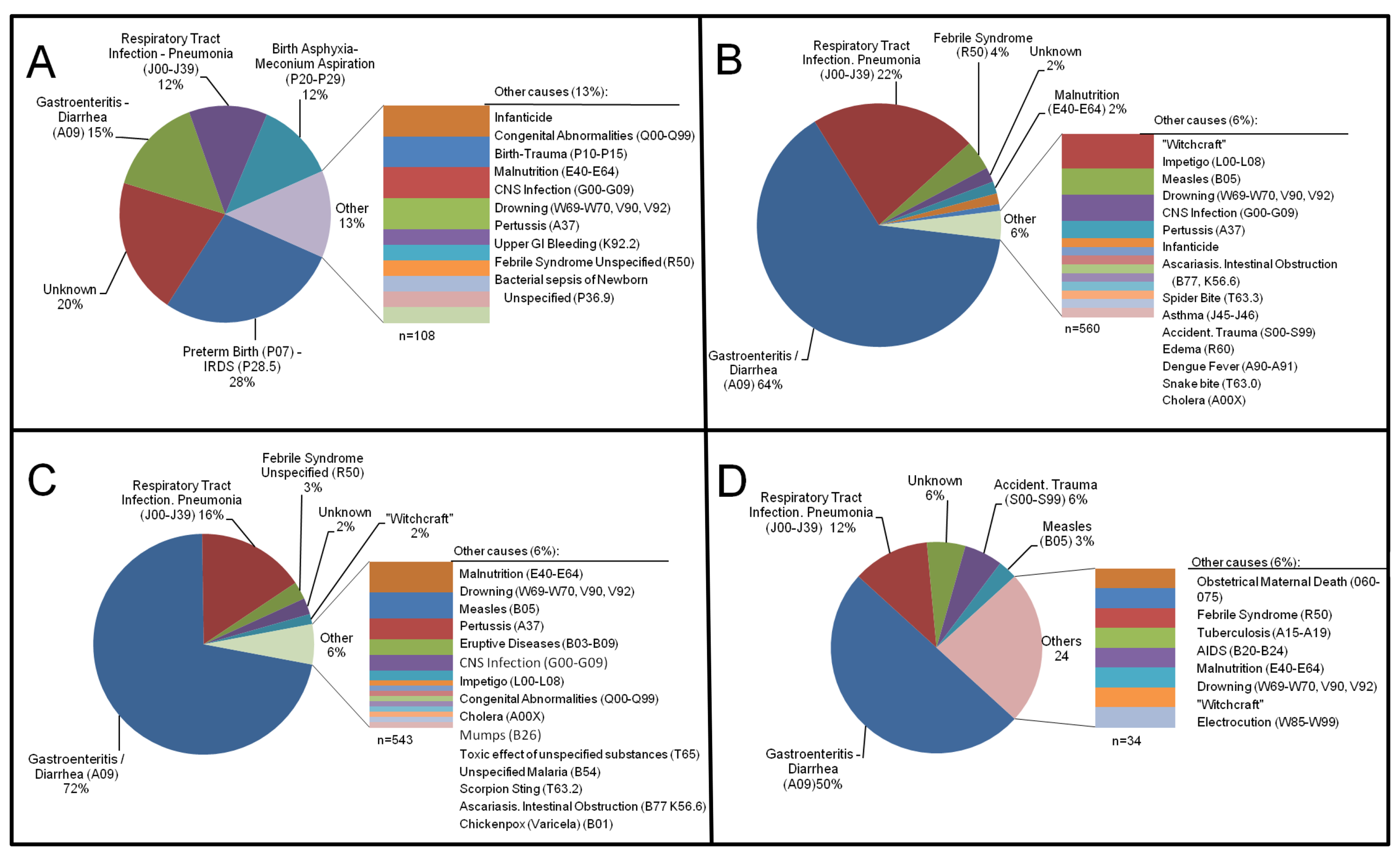

Supplement: Figure S2 — Causes of Death in Warao Children regarding different age groups. Neonates, <28 Days (A), Post-neonatal infants, 1-11 months (B), Toddlers and Preschool Children, 1-5 years (C), and children between 5 and 12 years (D). Causes of death were classified according to the International Classification of Diseases (ICD-10)24, although in some conditions the reported cause of death was unlikely to be the cause. Codes for diseases are shown inside parentheses. (TIF) [file pone.0085638.s004.tif]
