# Supplementary material for: Low Child Survival Index in a Multi-Dimensionally Poor Amerindian Population in Venezuela
Source: PLoS One. 2013 Dec 31;8(12):e85638. doi: 10.1371/journal.pone.0085638 (PMC3877389; doi:10.1371/journal.pone.0085638)
Supplement: Methods S1 — The detailed methods of sample selection (communities and houses selection) are described in Methods S1. (DOC) [file pone.0085638.s005.doc]

**Methods S1**

**DETAILED METHODS OF SAMPLE SELECTION**

**Communities’ selection:**

The list of communities appearing in the last official Venezuelan census (2001),^4^ was used as the sample-frame for each municipality. Pieces of paper with the names of all the communities on the census list (261) were placed in three boxes, each box representing one of the three municipalities, and one-hundred communities were selected at random. The proportion of communities selected from each municipality corresponded to the percentage of total communities within the municipality (table S1). The proportion slightly changed in the field because it was not possible to locate two communities in Tucupita and three in Antonio Diaz Municipalities. Two additional communities were subsequently included in Pedernales Municipality because there were two instances in which a geographical location identified as a single community in the census was regarded by the inhabitants as two different communities, although there was no physical division between them.

**Houses’ selection:**

Within each community a systematic sampling technique was used to select houses to be interviewed. As no map of the dwellings in the communities was available, only the total population of each community registered in the 2001 Venezuelan census,^4^ all the houses were counted before sampling. The first interviewed house was chosen by a simple random sampling method and subsequent houses were selected according to the size of the community (Fig. 2), as defined by the number of houses rather than the population. The procedure for selecting houses asked to participate was as follows. There were two bags, one with strips written with either left or right, and a second with pieces of paper containing the “n” numbers 1 – 5. First, left or right was blindly chosen, then the “n” number. We then walked along the river bank to the left or right from where our boat was docked, and approached the “nth” house encountered. After interviewing this first house, we continued in the same direction and approached subsequent houses according to the size of the community. In communities with >50 houses we approached every 4^th^ house, in communities with 11-50 dwellings, every 3^rd^ house, in communities with 6-10 dwellings, every second house, and in communities with less than 5 dwellings, we just approached one blindly chosen house (one strip was taken from the bag).

in communities with less than 5 dwellings, we approached the house nearest to the first house.
