# Supplementary material for: Low Child Survival Index in a Multi-Dimensionally Poor Amerindian Population in Venezuela
Source: PLoS One. 2013 Dec 31;8(12):e85638. doi: 10.1371/journal.pone.0085638 (PMC3877389; doi:10.1371/journal.pone.0085638)
Supplement: Methods S2 — The detailed MPI definition used in this study is described in Methods S2. (DOC) [file pone.0085638.s006.doc]

**Methods S2:**

**DETAILED MPI DEFINITION**

The Multidimensional Poverty Index (MPI) was used to classify the households as deprived across the three dimensions of the Human Development Index (Education, Health and Living Standards). The original description of the MPI^25,26^ was slightly modified as follow:

The **Education Dimension** included two indicators: Years of Schooling and Child School attendance. Each indicator was weighted equally at 1/6 (0.167).

-Years of Schooling: A woman was considered deprived if she or her husband/concubine/partner did not complete Primary school or basic education of the Venezuelan education system (grades 1 through 6).

-Child School attendance: : A woman was considered deprived if she lived in a household in which any school-aged child (7-14 years) was no attending to Primary School.

We just assessed the indicator Child Mortality of the **Health Dimension**. The indicator “Nutritional status” was not assessed in the study. The indicator was weighted at 1/6 (0.167).

-Child Mortality: A woman was considered deprived if any of her children had died

The **“Living Standards” Dimension** included six different indicators. Each indicator was weighted equally at 1/18 (0.056).

-Electricity: A woman was considered deprived if she lived in a household that was not connected to the fossil-fuel power plant of the community or did not have a electric portable generator.

-Sanitation: A woman was considered deprived if she lived in a household without improved sanitation (Public or shared latrine, Flush/pour flush to elsewhere –not into a pit, septic tank or sewer --, Open pit latrine, not connected to a piped sewer system)

-Drinking water: A woman was considered deprived if she lived in a household without access to safe drinking water (piped water into dwelling, public tap) or if she referred drinking water from the river without any treatment.

-Floor: A woman was considered deprived if her household had a dirt, sand or dung floor

-Cooking fuel: A woman was considered deprived if she did not have a gas cooker and used to cook with wood or charcoal.

-Assetts: A woman was considered deprived if her household had not more than one outboard motor or a handcrafted canoe. Missing data in the “Assets” indicator were assigned a zero value.

A woman was considered multidimensionally poor if she was deprived in at least 33.3% of the weighted indicators (MPI ≥0.333). A value of zero means no deprivation in all the indicators and 1 means deprivations in all of them.

The general MPI of the sample was calculated as Follows:

MPI= H x A

H: Percentage of women who were MPI poor (prevalence of poverty)

A: Average intensity of MPI poverty across the MPI-poor women.
