# Supplementary material for: Low Child Survival Index in a Multi-Dimensionally Poor Amerindian Population in Venezuela
Source: PLoS One. 2013 Dec 31;8(12):e85638. doi: 10.1371/journal.pone.0085638 (PMC3877389; doi:10.1371/journal.pone.0085638)
Supplement: Table S1 — Number and proportions of communities selected among different municipalities. (DOC) [file pone.0085638.s007.doc]

**Table S1**. Number and proportions of communities selected among different municipalities

| Municipalities | Communities selected (%) | Total Communities (%)* | Original goal (number of communities) |
| --- | --- | --- | --- |
| Pedernales | 14 (14.4) | 30 (11.5) | 12 |
| Tucupita | 10 (10.3) | 32 (12.3) | 12 |
| Antonio Diaz | 73 (75.3) | 199 (76.2) | 76 |
| **Total** | 97 (100) | 261 (100) | 100 |

*Venezuela’s National Census (2011) was used as a reference.
