# Supplementary material for: Low Child Survival Index in a Multi-Dimensionally Poor Amerindian Population in Venezuela
Source: PLoS One. 2013 Dec 31;8(12):e85638. doi: 10.1371/journal.pone.0085638 (PMC3877389; doi:10.1371/journal.pone.0085638)
Supplement: Table S2 — List of communities selected according to geographic location (Municipality). (DOC) [file pone.0085638.s008.doc]

**Table S2. List of communities selected according to geographic location (Municipality)**

| **Municipalities** | **Antonio Diaz** | | | **Pedernales** | **Tucupita** |
| --- | --- | --- | --- | --- | --- |
| **Communities** | -Kabiajoko | -Isla de Barril | -Abuenekabanoco | -Isla del Muerto | -Cojina II |
|  | -Burojoida | -Jobotoboto | -Ildamananoco | -Winamorena I / | -Nabasanuka |
|  | -Dajumana | -Juaneida de | -Bocoboco | -Winamorena II | de Makareo |
|  | -Hanakasi | Guayo | -Jotajana II | -Isla de Conoto | -Isla de |
|  | -Wanoko | -Jaruina | -Jotajana I | -Kajirina | Tucupita |
|  | -Jarijidoina | -Komaro | -Boca de | -Morocoto | -La Lagunita de |
|  | -Ibute | -Barakataina I y II | Nabakojoida | -Wakajara de | Mariusa |
|  | -Boca de Cojina | -Ajimuruina | -Nabakojoida | Manamo | -Punta Pescador |
|  | -Ajanocoida | -Jimuruina | -Guayaboroina de | -Boroma Janoko | -Kokoina |
|  | -Jioruina | -Batokonoko | Curiapo | (Barrio Chino) | -Mariusa |
|  | -Joroeneina | -Nayara | -Moraina de Curiapo | -La Culebrita | -Yacariyene |
|  | -Jimurina | -Santa Rosa de | -Moraina | -Isla Misteriosa | -Janokosebe |
|  | -Isla de Buari | Guayo | -Osibukabinoko | -Najakaina | -Volcán/Puerto |
|  | -Usidu | -Jokabanoko I | -Ajotejana | -Jagüey de los Cocos | de Volcán |
|  | -Isla de Jobure | -Jokabanoko II | -Curiapo | -Simoina |  |
|  | -Cocoina | -Moninoko | -Kaiwire | -Kajirina |  |
|  | -Idoburojo | -Tekoburojo | -Jotasanuka | -Waranoko II |  |
|  | -Caño Yeri | -Guayaboroina | -Barakaro |  |  |
|  | -Guajakajisi | -Campo Alegre | -Kuarejoro |  |  |
|  | -Yaguara | -La Mora | -Mojabaina de |  |  |
|  | -Jeukubaka | -Kuamujo | Bonoina |  |  |
|  | -Sarewabanoko I | -Jerisabanoko | -Burojosanuka |  |  |
|  | -Sarewabanoko II | -Jobure de Guayo | -Jobasujuru |  |  |
|  | -Jotabuidanoko | -Merejina | -Musimurina |  |  |
|  | -Tobewabanoko | -Murako | -Siawani |  |  |
|  |  | -San Francisco de | -Nabasanuka |  |  |
|  |  | Guayo | -Bonoina |  |  |
