# Supplementary material for: Low Child Survival Index in a Multi-Dimensionally Poor Amerindian Population in Venezuela
Source: PLoS One. 2013 Dec 31;8(12):e85638. doi: 10.1371/journal.pone.0085638 (PMC3877389; doi:10.1371/journal.pone.0085638)
Supplement: Table S3 — Distribution of the sample regarding size of the evaluated communities. (DOC) [file pone.0085638.s009.doc]

**Table S3. Distribution of the sample regarding size of the evaluated communities.**

| **Categories (Number of Houses)** | **Frequency (%)** |
| --- | --- |
| 1 (1-10) | 161 (23.4) |
| 2 (11-25) | 134 (19.5) |
| 3 (26-50) | 175 (25.4) |
| 4 (51-100) | 103 (15) |
| 5 (>100) | 115 (16.7) |

Categories 1 through 5 correspond to the number of houses per community (in parentheses).
