# Supplementary material for: Low Child Survival Index in a Multi-Dimensionally Poor Amerindian Population in Venezuela
Source: PLoS One. 2013 Dec 31;8(12):e85638. doi: 10.1371/journal.pone.0085638 (PMC3877389; doi:10.1371/journal.pone.0085638)
Supplement: Table S4 — Variables included in the different models. (DOC) [file pone.0085638.s010.doc]

**Table S4.** Variables included in the different models

| **Independent Variables** | **Type** | **Levels** | **Multivariable** | **Multivariable** | **PROC** | **PROC** |
| --- | --- | --- | --- | --- | --- | --- |
|  |  |  | **Linear** | **Linear** | **GLM** | **GLM** |
|  |  |  | **Regression 1** | **Regression 2** | **Model 1** | **Model 2** |
| Municipality | Categorical | 3 | x |  | x | x |
| Doctor’s Presence | Dummy | 2 | x | x | x | x |
| Maternal Age | Continuous | - | x | x | x | x |
| Mother’s Literacy | Dummy | 2 | x | x | x | x |
| Profession of the household head  (teacher/nurse vs. others) | Dummy | 2 | x | x | x | x |
| Presence of elementary school | Dummy | 2 | x | x | x | x |
| Presence of primary health care facility | Dummy | 2 | x | x | x | x |
| Number of People living in house | Continuous | - | x | x | x | x |
| Presence of water treatment plant | Dummy | 2 | x | x | x | x |
| Number of houses per community | Continuous | - | x | x | x | x |
| Parity | Continuous | - |  |  | x | x |
| Multidimensional Poverty Index | Continuous | - |  |  | x | x |
| Father’s presence in household | Dummy | 2 |  |  |  | x |
| Dwelling characteristics | Dummy | 2 |  |  |  | x |
| (traditional construction vs. not traditional) |  |  |  |  |  |  |
| Presence of religious mission | Dummy | 2 | x | x |  | x |
| Presence of water treatment practices | Dummy | 2 | x | x |  |  |
| Presence of an outboard motor | Dummy | 2 | x | x |  |  |
| Main water source (river water) | Dummy | 2 | x | x |  |  |
| Subregions | Categorical |  |  | x |  |  |
