# Supplementary material for: Low Child Survival Index in a Multi-Dimensionally Poor Amerindian Population in Venezuela
Source: PLoS One. 2013 Dec 31;8(12):e85638. doi: 10.1371/journal.pone.0085638 (PMC3877389; doi:10.1371/journal.pone.0085638)
Supplement: Table S5 — Characteristics of communities among different geographical regions (Basic Services). (DOC) [file pone.0085638.s011.doc]

**Table S5.** **Characteristics of communities among different geographical regions (Basic Services).**

| **Subregion** | **Communities without access to** | | | | | | | | | | | | | | |
| --- | --- | --- | --- | --- | --- | --- | --- | --- | --- | --- | --- | --- | --- | --- | --- |
|  | Elementary | | High | | Religious | | Fossil-Fuel | | Water-Treatment | | Sewage | | Primary | | Medical |
|  | School (%) | | School (%) | | Mission (%) | | Power Plant (%) | | Plant (%) | | System (%) | | Healthcare | | Doctor (%) |
|  |  | |  | |  | |  | |  | |  | | Institute (%) | |  |
| Upper Delta | 0 | | 4 (100) | | 3 (75) | | 0 | | 3 (75) | | 4 (100) | | 2 (50) | | 3 (75) |
| Mariusa Atlantic | 4 (67) | | 6 (100) | | 5 (83) | | 5 (83) | | 6 (100) | | 6 (100) | | 6 (83) | | 6 (100) |
| Coastline- |  | |  | |  | |  | |  | |  | |  | |  |
| Makareo |  | |  | |  | |  | |  | |  | |  | |  |
| Distributary |  | |  | |  | |  | |  | |  | |  | |  |
| Guayo | 34 (71) | | 47 (98) | | 44 (92) | | 42 (88) | | 44 (92) | | 48 (100) | | 45 (94) | | 47 (98) |
| surroundings |  | |  | |  | |  | |  | |  | |  | |  |
| Curiapo | 12 (80) | | 14 (93) | | 13 (87) | | 11 (73) | | 14 (93) | | 15 (100) | | 12 (80) | | 14 (93) |
| surroundings |  | |  | |  | |  | |  | |  | |  | |  |
| Nabasanuka | 2 (20) | | 8 (80) | | 8 (80) | | 4 (40) | | 8 (80) | | 10 (100) | | 7 (70) | | 9 (90) |
| surroundings |  | |  | |  | |  | |  | |  | |  | |  |
| Manamo | 3 (33) | | 8 (89) | | 9 (100) | | 7 (78) | | 9 (100) | | 9 (100) | | 5 (56) | | 8 (89) |
| Distributary |  | |  | |  | |  | |  | |  | |  | |  |
| Capure | 2 (40) | | 5 (100) | | 5 (100) | | 5 (100) | | 5 (100) | | 5 (100) | | 5 (100) | | 5 (100) |
| distributary- |  | |  | |  | |  | |  | |  | |  | |  |
| Waranoko |  | |  | |  | |  | |  | |  | |  | |  |
| Surroundings |  | |  | |  | |  | |  | |  | |  | |  |
| **Municipality** |  | | | | | | | | | | | | | | |
| Pedernales | 5 (36) | 13 (93) | | 14 (100) | | 12 (86) | | 14 (100) | | 14 (100) | | 10 (71) | | 13 (93) | |
| Tucupita | 4 (40) | 10 (100) | | 8 (80) | | 5 (50) | | 9 (90) | | 10 (100) | | 8 (80) | | 9 (90) | |
| Antonio Diaz | 48 (66) | 69 (95) | | 65 (89) | | 57 (78) | | 66 (90) | | 73 (100) | | 64 (88) | | 70 (96) | |
| **TOTAL** | 57 (59) | 92 (95) | | 87 (90) | | 74 (76) | | 89 (92) | | 97 (100) | | 82 (85) | | 90 (93) | |
