# Supplementary material for: Low Child Survival Index in a Multi-Dimensionally Poor Amerindian Population in Venezuela
Source: PLoS One. 2013 Dec 31;8(12):e85638. doi: 10.1371/journal.pone.0085638 (PMC3877389; doi:10.1371/journal.pone.0085638)
Supplement: Table S6 — Distribution of sample and child survival-Index among different subregions of the Orinoco Delta. (DOC) [file pone.0085638.s012.doc]

**Table S6. Distribution of sample and child survival-Index among different subregions of the Orinoco Delta**

| **Geographical Subregion** | **Frequency (%)** | **Total Female** | **% Represented by** | **Dead** | **Child Survival-Index** |
| --- | --- | --- | --- | --- | --- |
|  |  | **Population^4^** | **sample (*95% CI)*** | **children (%)** | **(Mean ± SD)** |
| Curiapo surroundings | 93 (13.5) | 1388 | 6.7 (5.5-8.1) | 27.7 | 74.6 ± 26 |
| Guayo surroundings | 333 (48.4) | 3231 | 10.3 (9.3-11.4) | 30.7 | 74.4 ± 26 |
| Nabasanuka surroundings | 73 (10.6) | 2604 | 2.8 (2.2-3.5) | 25.8 | 80.4 ± 22 |
| Mariusa Atlantic Coastline- | 52 (7.6) | 612 | 8.5 (6.5-10.9) | 48.4 | 61.2 ± 32 |
| Makareo Distributary |  |  |  |  |  |
| Upper Delta | 39 (5.7) | 124 | 31.4 (23.9-40.1) | 24.7 | 80.4 ± 20 |
| Manamo Distributary | 68 (9.9) | 926 | 7.3 (5.8-9.2) | 32.7 | 72.9 ±23 |
| Capure Distributary-Waranoko | 30 (4.4) | 420 | 7.1 (5.1-10) | 39.9 | 65.3 ±25 |
| Surroundings |  |  |  |  |  |
| TOTAL | 688 (100) | 9305 | 7.4 (6.9-7.9) | 31.6 | 73.8 ± 26 |
