# Supplementary material for: Low Child Survival Index in a Multi-Dimensionally Poor Amerindian Population in Venezuela
Source: PLoS One. 2013 Dec 31;8(12):e85638. doi: 10.1371/journal.pone.0085638 (PMC3877389; doi:10.1371/journal.pone.0085638)
Supplement: Table S7 — Multivariable linear regression analysis. Model 2: Association of CSI with socioeconomic factors. (DOC) [file pone.0085638.s013.doc]

**Table S7. Multivariable linear regression analysis. Model 2: Association of CSI with socioeconomic factors**

| **Variables** | **Subcategory** | **Children Survival** | | |
| --- | --- | --- | --- | --- |
|  |  | **Coeff (SE) †** | ***T* value** | ***P* value** |
| **Mother’s Place of Residence** |  |  | | |
|  | Upper Orinoco Delta (A) | Reference | | |
|  | Manamo Distributary (B) | -0.017 (0.06) | -0.301 | 0.76 |
|  | Capure distributary- Waranoko | -0.031 (0.07) | -0.464 | 0.64 |
|  | surroundings (C) |  |  |  |
|  | Mariusa Atlantic Coastline – Makareo | -0.11 (0.06) | -1.766 | 0.07 |
|  | Distributary (D) |  |  |  |
|  | Nabasanuka surroundings (E) | 0.02 (0.05) | 0.391 | 0.69 |
|  | Guayo surroundings (F) | -0.008 (0.05) | -0.163 | 0.87 |
|  | Curiapo surroundings (G) | 0.029 (0.05) | 0.524 | 0.6 |
| **Mother’s Characteristics** |  |  |  |  |
|  | Age | -0.006 (0.0008) | -7.107 | <0.0001* |
|  | Illiteracy | -0.062 (0.02) | -2.649 | 0.008* |
| **Living in a community** |  |  |  |  |
| **without access to** |  |  |  |  |
|  | Elementary school | -0.059 (0.03) | -1.846 | 0.06 |
|  | Primary healthcare institution | -0.022(0.04) | -0.531 | 0.59 |
|  | Water treatment plant | -0.07 (0.04) | -1.738 | 0.08 |
|  | Medical doctor | -0.027 (0.04) | -0.645 | 0.51 |
|  | Religious mission | 0.002 (0.04) | 0.056 | 0.96 |
| **Size of the Community** | | 0.019 (0.01) | 1.469 | 0.14 |
| **Household Characteristics** |  |  |  |  |
|  | Profession of the household head | -0.14 (0.04) | -3.415 | 0.0007* |
|  | (being other than nurse or teacher) |  |  |  |
|  | Number of People per house | 0.002 (0.002) | 1.258 | 0.21 |
|  | Absence of water treatment practices | 0.02 (0.03) | 0.643 | 0.52 |
| **Others** |  |  |  |  |
|  | Presence of an outboard motor | 0.02 (0.02) | 1.041 | 0.29 |
|  | Main water source (river water) | -0.003 (0.03) | -0.138 | 0.89 |

**†** Negative sign (coefficient) denotes inverse association of the independent variable with CSI

*Statistically significant *p*<0.05

Residual standard error: 0.2406 on 624 degrees of freedom (51 observations deleted due to missingness)

Multiple R-squared: 0.1777 / Adjusted R-squared: 0.1527

F-statistic: 7.1 on 19 and 624 DF / p-value: < 2.2e-16
