# Supplementary material for: Low Child Survival Index in a Multi-Dimensionally Poor Amerindian Population in Venezuela
Source: PLoS One. 2013 Dec 31;8(12):e85638. doi: 10.1371/journal.pone.0085638 (PMC3877389; doi:10.1371/journal.pone.0085638)
Supplement: Table S8 — Generalized Linear Model predicting determinants of Child Survival Index (CSI). Model 1. (DOC) [file pone.0085638.s014.doc]

**Table S8. Generalized Linear Model predicting determinants of Child Survival Index (CSI). Model 1**

| **Variables** |  | **Degrees of Freedom** | **F Statistic** | ***p*-value** |
| --- | --- | --- | --- | --- |
| **Municipality of residence** | | 2 | 0.11 | 0.89 |
| **Mother’s Characteristics** |  |  |  |  |
|  | Age | 1 | 16.26 | <0.0001* |
|  | Illiteracy | 1 | 0.19 | 0.66 |
|  | Parity | 1 | 3.45 | 0.06 |
| **Living in a community** |  |  |  |  |
| **without access to** |  |  |  |  |
|  | Elementary School | 1 | 2.8 | 0.09 |
|  | Primary Healthcare facility | 1 | 0.38 | 0.54 |
|  | Water Treatment Plant | 1 | 1.75 | 0.19 |
|  | Medical Doctor | 1 | 0.92 | 0.34 |
| **Size of the community (number of houses)** | | 1 | 1.37 | 0.24 |
| **Household Characteristics** |  |  |  |  |
|  | Profession of the household head | 1 | 4.09 | 0.04* |
|  | (being other than nurse or teacher) |  |  |  |
|  | Number of People per house | 1 | 3.07 | 0.08 |
|  | MPI | 1 | 155.59 | <0.0001* |

*Statistically significant. *p*-value <0.05
