# Supplementary material for: Low Child Survival Index in a Multi-Dimensionally Poor Amerindian Population in Venezuela
Source: PLoS One. 2013 Dec 31;8(12):e85638. doi: 10.1371/journal.pone.0085638 (PMC3877389; doi:10.1371/journal.pone.0085638)
Supplement: Table S9 — Model Fitness Information of generalized linear model procedures. (DOC) [file pone.0085638.s015.doc]

**Table S9. Model Fitness Information of generalized linear model procedures.**

**Generalized linear m**odel 1

| **Source** | **Degrees of freedom** | **Sum of Squares** | **Mean Square** | **F Value** | **p-value** |
| --- | --- | --- | --- | --- | --- |
| **Model** | 13 | 16.05509065 | 1.23500697 | 27.66 | <.000000000000000001 |
| **Error** | 674 | 30.09085220 | 0.04464518 |  |  |
| **Corrected Total** | 687 | 46.14594285 |  |  |  |

| **R-Square** | **Coeff Var** | **Root MSE** | **CSI Mean** |
| --- | --- | --- | --- |
| 0.347920 | 28.61378 | 0.211294 | 0.738435 |

**Generalized linear model** 2

| **Source** | **Degrees of freedom** | **Sum of Squares** | **Mean Square** | **F Value** | **p-value** |
| --- | --- | --- | --- | --- | --- |
| **Model** | 16 | 16.51323922 | 1.03207745 | 23.37 | <.000000000000000001 |
| **Error** | 671 | 29.63270363 | 0.04416200 |  |  |
| **Corrected Total** | 687 | 46.14594285 |  |  |  |

| **R-Square** | **Coeff Var** | **Root MSE** | **CSI Mean** |
| --- | --- | --- | --- |
| 0.357848 | 28.45852 | 0.210148 | 0.738435 |
